# Airway Abnormalities in Adult Mucopolysaccharidosis and Development of Salford Mucopolysaccharidosis Airway Score

**DOI:** 10.3390/jcm10153275

**Published:** 2021-07-24

**Authors:** Chaitanya Gadepalli, Karolina M. Stepien, Reena Sharma, Ana Jovanovic, Govind Tol, Andrew Bentley

**Affiliations:** 1 Ear Nose and Throat Department, Salford Royal NHS Foundation Trust, Manchester M6 8HD, UK; 2 Adult Inherited Metabolic Department, Salford Royal NHS Foundation Trust, Manchester M6 8HD, UK; karolina.stepien@srft.nhs.uk (K.M.S.); reena.sharma@srft.nhs.uk (R.S.); ana.jovanovic@srft.nhs.uk (A.J.); 3 Anaesthetics Department, Salford Royal NHS Foundation Trust, Manchester M6 8HD, UK; govind.tol@srft.nhs.uk; 4 Intensive Care & Respiratory Medicine, Manchester University NHS Foundation Trust, Wythenshawe Hospital, Manchester M23 9LT, UK; andrew.bentley@mft.nhs.uk

**Keywords:** mucopolysaccharidoses, airway, obstruction, management

## Abstract

(1) Background: Mucopolysaccharidoses (MPS) are a heterogeneous group of lysosomal storage disorders caused by the absence of enzymes required for degradation of glycosaminoglycans (GAGs). GAGs deposition in tissues leads to progressive airway narrowing and/or tortuosity. Increased longevity of patients has posed newer problems, especially the airway. This study aims to characterise various airway abnormalities in adult MPS from a regional centre and proposes a method to quantify the severity of the airway disease. (2) Methods: Retrospective analysis by case notes review, clinical examination, endoscopy, cross-sectional imaging, 3-dimensional reconstruction, and physiological investigations were used to assess the airway abnormalities. Quantitative assessment of the airway severity was performed a validated questionnaire of 15 parameters to derive Salford Mucopolysaccharidosis Airway Score (SMAS). (3) Results: Thirty-one adult MPS patients (21M/ 9F; median 26.7 years; range 19–42 years) were reviewed. There were 9 MPS I, 12 MPS II, 2 MPS III, 5 MPS IV, 2 MPS VI, and 1 MPS VII. Airway abnormalities in each MPS type are described. Patients scoring more than 35 on SMAS had some form of airway intervention. The area under curve of 0.9 was noted at a score of 25, so SMAS more than 25 may predict a difficult airway and potential to have complications. Pearson’s correlation between SMAS and height, weight, BMI were poor (*p* < 0.05). (4) Conclusions: Airway abnormalities in adult MPS are varied and complex. Assessment of the airway should be holistic and include multiple parameters. An objective multidimensional score such as SMAS may help to predict and manage difficult airways warranting further investigation and validation.

## 1. Introduction

Mucopolysaccharidoses (MPS) are rare, inherited, lysosomal storage diseases with a combined incidence of 1 in 22,000 [1]. Lysosomal hydrolase enzyme deficiencies result in accumulation of glycosaminoglycans (GAGs), leading to structural abnormalities and organ dysfunction that can increase the risk of anaesthesia complications [2]. Depending on the type of MPS, glycosaminoglycan accumulations can occur in various organs, resulting in cardiovascular, pulmonary, gastrointestinal, neurologic, and musculoskeletal dysfunction (Table 1) [3]. MPS can be grouped into four broad categories according to their dominant clinical features: (1) MPS I, II, and VII affect soft tissue storage and the skeleton with or without intracranial involvement; (2) MPS VI affects both soft tissues and the skeleton; (3) MPS IVA and IVB are primarily associated with skeletal disorders; and (4) MPS III A–D primarily affects the central nervous system [4].

GAG accumulation in the upper airway results in hypertrophy of soft tissues, including adenoids, tonsils, tongue, and laryngopharynx, which may all cause airway problems and pose difficulty in anaesthetic airway management due to bulky airways. This is especially important because MPS patients frequently require surgical interventions requiring anaesthesia [4]. Airway complications are a common feature of MPS I, II, IV, and VI and considerably contribute to morbidity and premature mortality [7,8]. The bulky airways can predispose to breathing related problems, which may worsen during sleep. These include snoring, upper airway resistance syndrome, obstructive sleep apnoea [9]. A multidimensional assessment by Dalewski et al. [10], which incorporates modified Mallampati score [11], upper airway volume measurements using CT scan, Berlin questionnaire [12], is useful. Measures of airway obstruction and pulmonary function have frequently been used as primary or secondary outcomes in interventional trials [3,13,14,15]. Current therapeutic modalities, such as enzyme replacement therapy (ERT) in MPS I Hurler–Scheie (HS) and Scheie, II, IVA, and VI, and haemopoietic stem cell transplantation (HSCT) in MPS I Hurler (H), have demonstrated organ specific and systemic metabolic correction [16,17,18,19]. Despite the positive outcomes, airway disease continues to cause significant complications resulting from structural rather than inflammatory abnormalities [20,21,22]. Current treatment with ERT fails to fully reverse adenotonsillar storage pathology in MPS type I, III, IV, and VI, manifesting with ongoing clinical disease [21]. It has been previously shown that pathological changes in tonsils and adenoids are responsible for not only the increased incidence of hypertrophy causing obstruction, but also the high regrowth rate in adenoid tissue commonly necessitating revision surgery later in adolescence and possibly adulthood [20,21]. Airway problems in MPS are multifactorial as evidenced by limited relief from adenotonsillectomy [8]. By the time an MPS patient has transitioned from paediatric to adult care, issues such as adenoids and tonsils may have already been addressed, and the airway problems shift towards other aspects of upper and lower airways.

We aim to evaluate and characterise airway abnormalities in adult MPS patients. The purpose of this study is to identify various airway abnormalities in our cohort of adult MPS and quantify the degree of airway problems for prognosis and planning. We have used various parameters that can adversely affect the airway and developed Salford MPS Airway Score (SMAS) as a novel tool in assessing the severity of airway disease in adult MPS disorders.

## 2. Materials and Methods

### 2.1. Study Design

Retrospective review of case notes of adult MPS patients attending airway assessment was performed. Ethical approval from the Research and Innovation department, Salford Royal NHS Foundation Trust, Northern Care Alliance NHS, United Kingdom was obtained, reference: S20HIP40.

### 2.2. Patients

All patients were assessed in airway multi-disciplinary team clinic by the same anaesthetic and ear, nose, and throat consultant with special interest in adult MPS airways. In our cohort, the specific modalities of treatment for MPS patients were enzyme replacement therapy (ERT), haematopoietic stem cell transplant (HSCT), or none.

### 2.3. Assessment

Apart from clinical examination, investigative tools such as nasendoscopy, computer tomography scans, pulmonary function tests, three-dimensional imaging, and virtual endoscopy were sought where possible. Nasendoscopy is performed by passing a fibre optic camera via the nasal cavity and examination of the nasal cavities, oropharynx, and larynx under a local anaesthetic.

### 2.4. SMAS

Fifteen parameters were chosen that can holistically assess both upper and lower airway. The parameters and method to calculate the SMAS are depicted in Table 2. Each of these parameters are graded in an ordinal score from zero to three: zero corresponding to normal, one—mild abnormality, two—moderate abnormality, and three—severe abnormality. Adding the score for each of the 15 parameters will provide a final score, which will quantify the degree of airway severity. A high score corresponds to a complex airway. The range of the score can be from 0 to 45. Parameters one to six are calculated using clinical examination. Protrusion of teeth and bulkiness of the tongue can be assessed on clinical examination and CT scans. Parameters 7 to 10 are calculated using nasendoscopy. Parameters 11 to 13 are calculated using cross-sectional imaging such as CT scans. Parameters 14 and 15 are calculated using pulmonary function tests. Certain parameters such as pulmonary function tests cannot be carried out in patients who lack capacity or would not comply with the assessment. Likewise, nasendoscopy cannot be carried without a patient’s co-operation. This will limit the maximal score that can be attained. The content, criterion validity, and clinical use of the questionnaire was assessed by distributing the questionnaire to 15 senior anaesthetists to be used in their daily practice. To assess the impact of body habitus on the airway, Pearson’s correlation was used to assess the relationship between height, weight, body mass index, and SMAS. To assess the usefulness of the SMAS score in clinical application, a receiver operating curve (ROC) curve was plotted.

## 3. Results

Thirty-one adult MPS patients were reviewed, there were 21 males and 10 females. The age range was 19–43 years. The mean age was 28 years, and the median age was 26.7 years. Table 3 shows the demographics in each type of MPS.

Various airway abnormalities were noted in the MPS patients. The modified Mallampati grade was either three or four in all patients. There was a pattern of macroglossia, high larynx, and large epiglottis noted in almost all patients in the cohort. MPS I and MPS III had the least severe airway abnormalities. In both these groups, learning difficulties and/or blindness was a relevant challenge. Common airway abnormalities and relevant airway challenges in each type of MPS are depicted in Table 4.

### 3.1. Nasendoscopy

**Figure 1:**
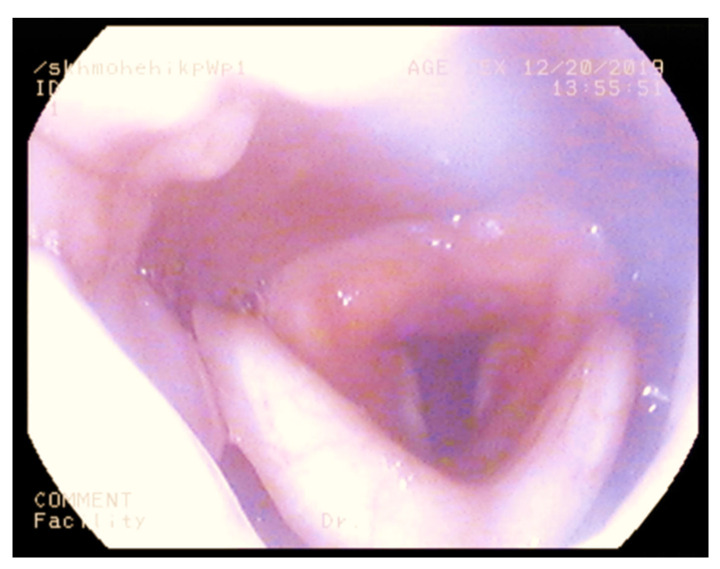
High anterior larynx in mucopolysacharridosis I.

**Figure 2:**
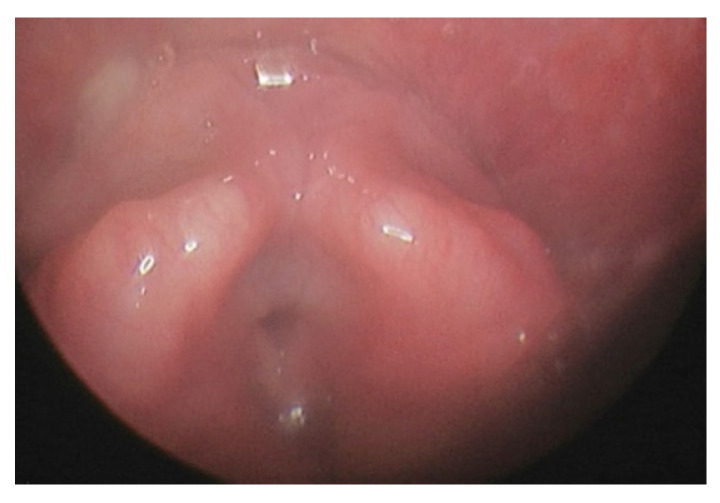
Bulky supraglottis in mucopolysacharridosis II.

**Figure 3:**
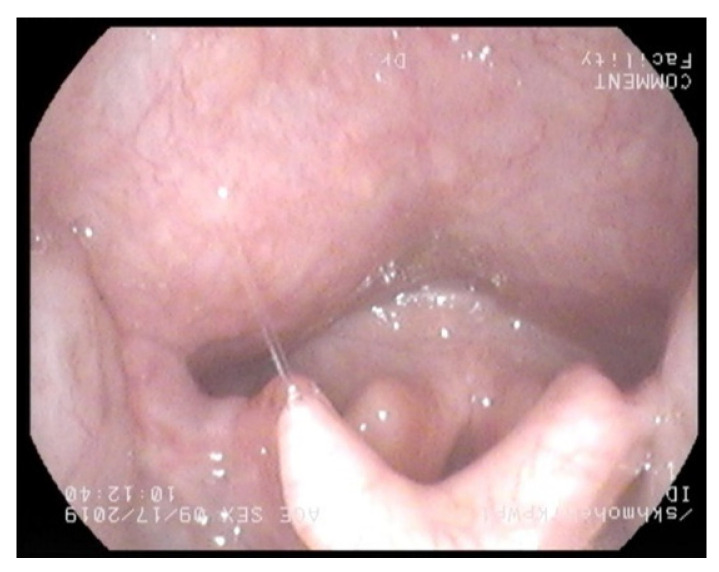
Large overhanging epiglottis, high larynx in mucopolysacharridosis IV.

**Figure 4:**
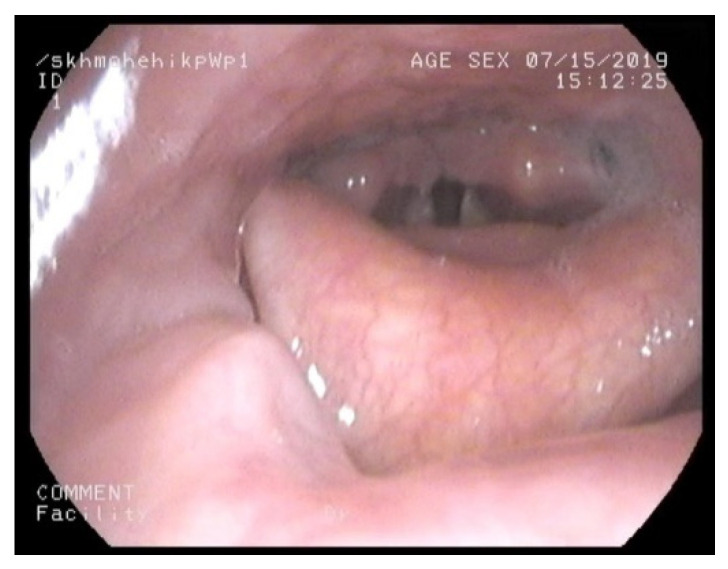
Large epiglottis almost touching soft palate in mucopolysacharridosis VI.

This outpatient procedure helped us to assess which nasal cavity was wider to plan nasal intubation, assess the height and bulk of epiglottis, bulk of posterior two-thirds of the tongue, appearance of supraglottis, and vocal cord mobility. It also provided information on oropharyngeal or supraglottic collapse on valsalva manoeuvre. A bulky supraglottis made use of supraglottic airway devices such as laryngeal mask airway or trans nasal humidified rapid insufflation ventilatory exchange (THRIVE) difficult. Figure 1, Figure 2, Figure 3 and Figure 4 shows a nasendoscopy appearances in various types of MPS.

### 3.2. Cross-Sectional Imaging

**Figure 5:**
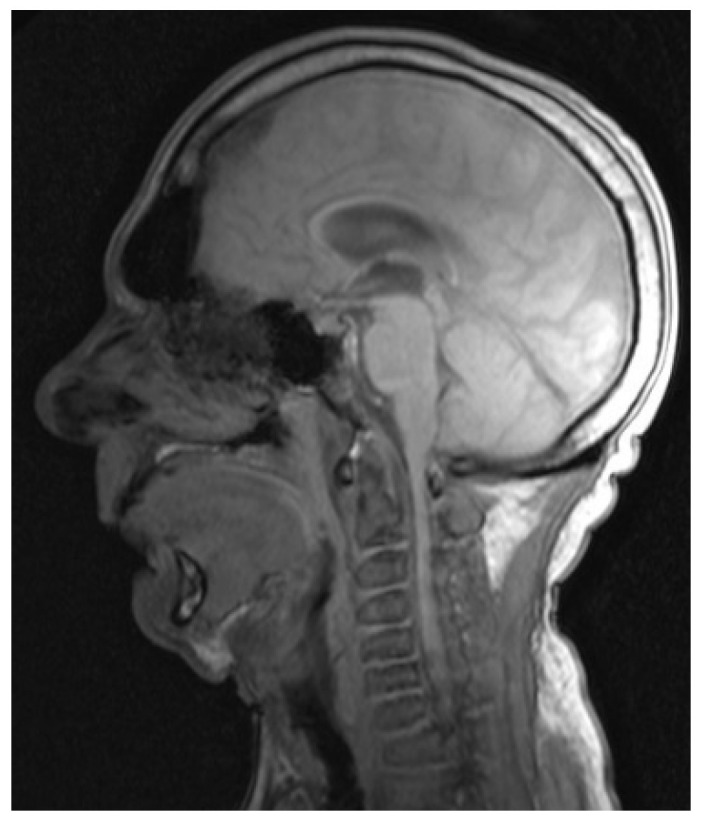
MRI scan of MPS I showing large tongue and high, anterior larynx, and absence of cervical lordosis.

**Figure 6:**
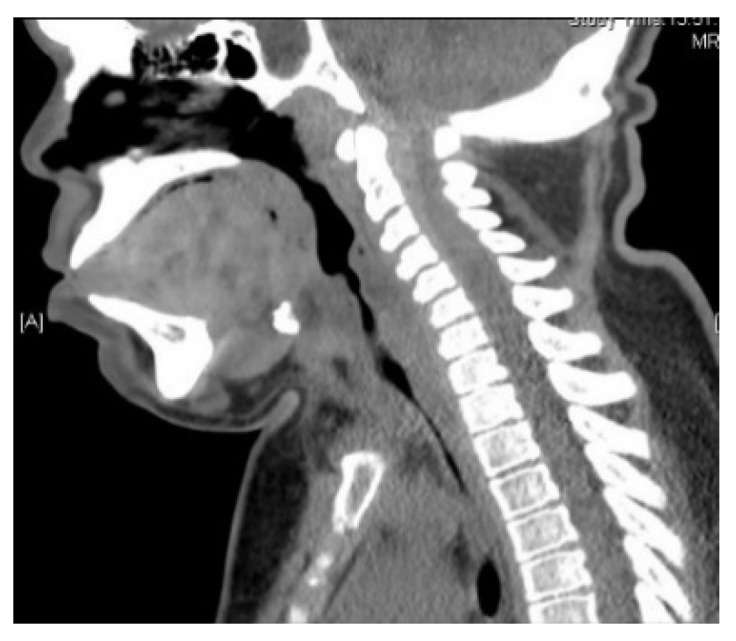
CT scan of MPS II, showing large bulky tongue, flat palate, prominent teeth, short neck, and absence of cervical lordosis.

**Figure 7:**
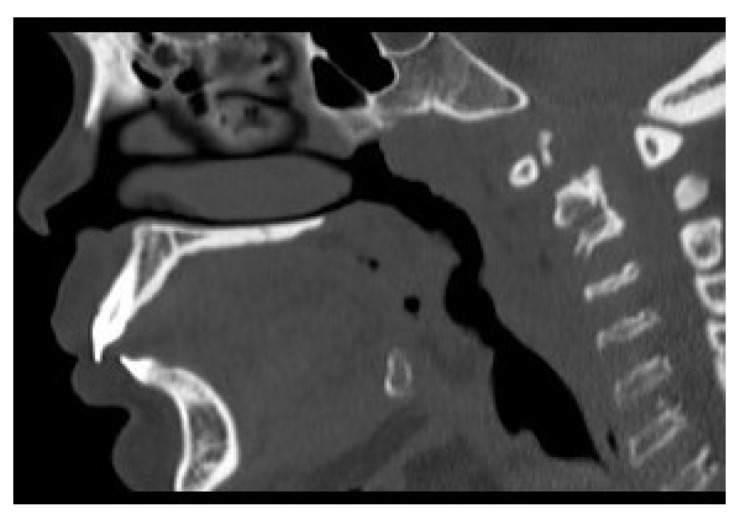
CT scan of MPS IV showing contracted nasopharynx, large tongue, prominent teeth, and high larynx.

**Figure 8:**
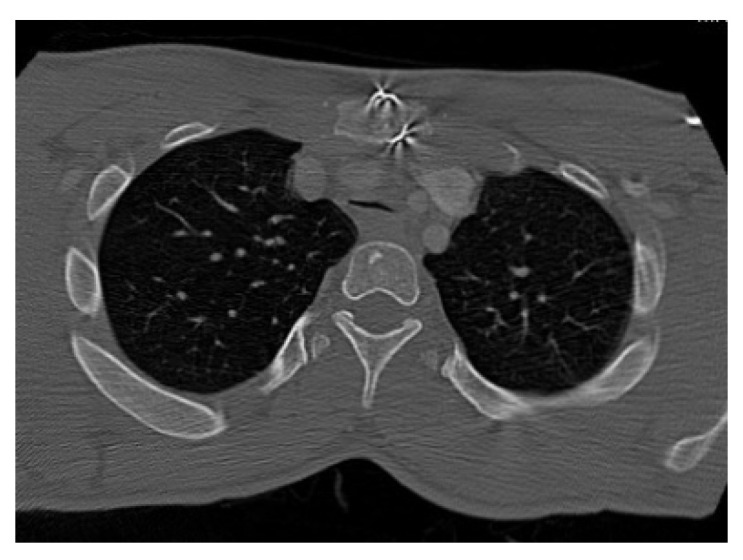
CT scan of MPS II showing collapsed trachea, suggestive of tracheomalacia.

Computer tomography (CT) and magnetic resonance imaging (MRI) are very helpful in assessing both upper and lower airway. In the upper airway, the bulky soft tissue of the tongue and supraglottis can be assessed using MRI scans. In the lower airway, the calibre of the airway, tracheomalacia, tracheal stenosis, and tracheal tortuosity can be assessed using CT scans. Figure 5, Figure 6, Figure 7 and Figure 8 shows cross-sectional imaging in various types of MPS.

### 3.3. 3-Dimensional Reconstruction (3D) and Virtual Endoscopy (VE)

**Figure 9:**
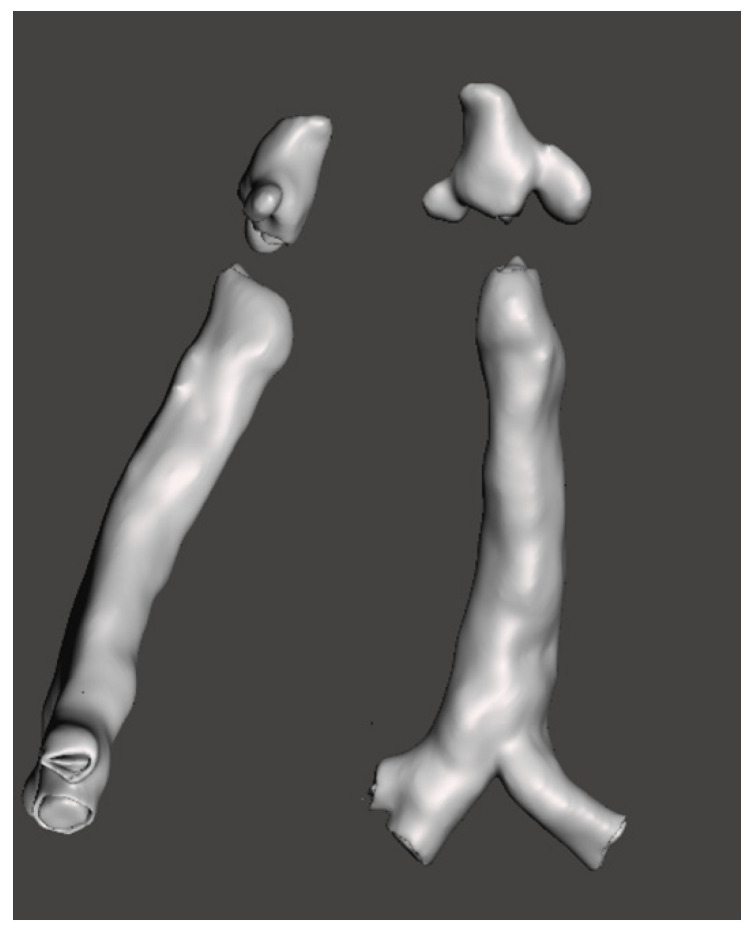
3-Dimensional reconstruction of MPS I showing normal trachea.

**Figure 10:**
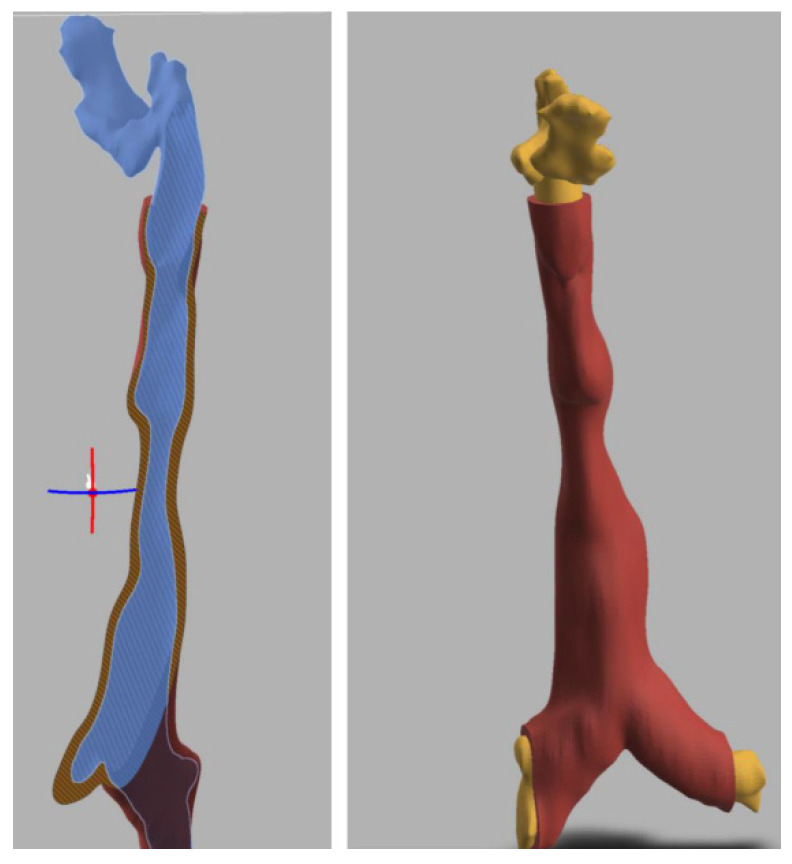
3-Dimensional reconstruction of MPS II showing tracheomalacia.

**Figure 11:**
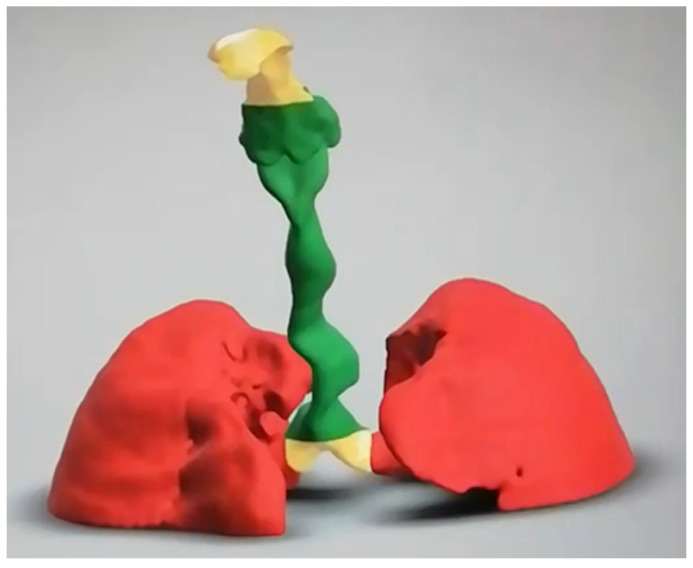
3-Dimensional reconstruction of MPS IV showing tortuous trachea and tracheomalacia.

**Figure 12:**
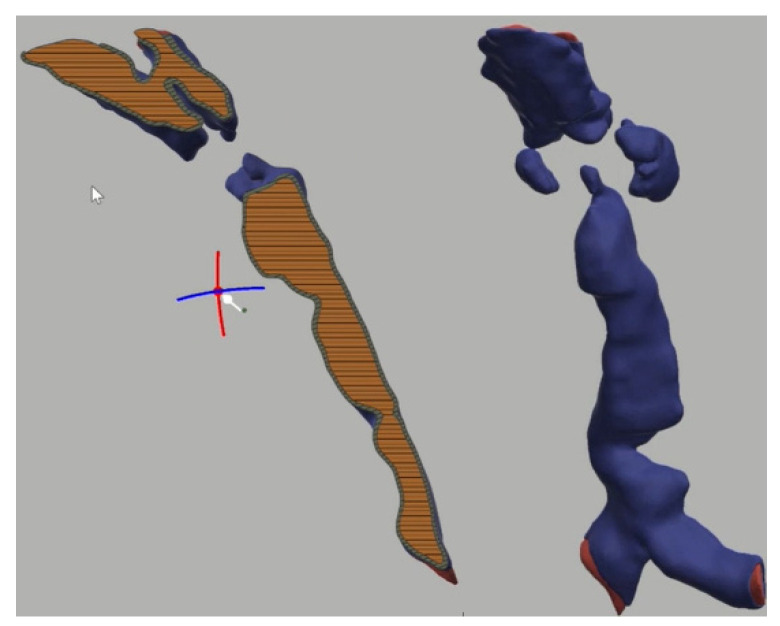
3-Dimensional reconstruction of MPS VI showing tortuous trachea and tracheomalacia.

**Figure 13:**
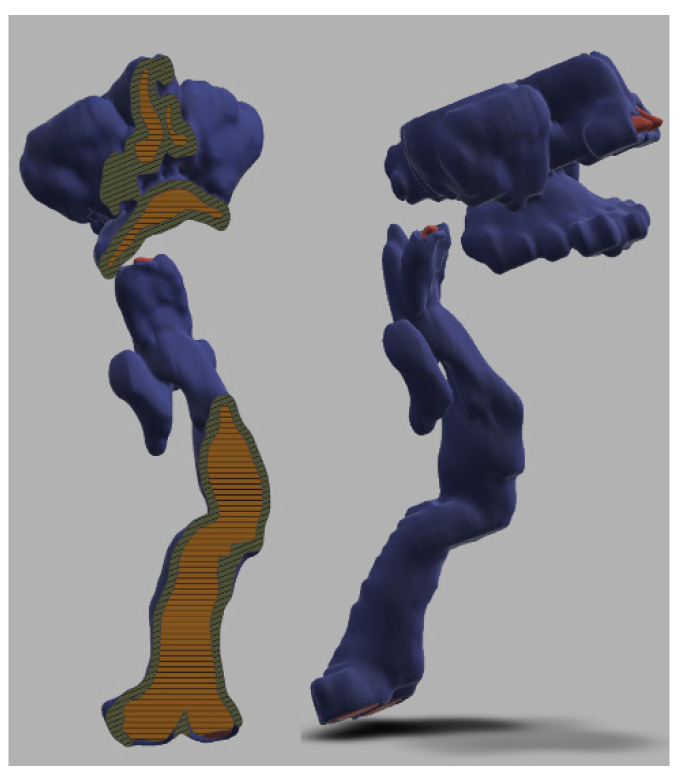
3-Dimensional reconstruction of MPS VII showing tortuous trachea and tracheomalacia.

Using information from the cross-sectional imaging, 3-dimensional reconstruction and virtual endoscopy of the airway can be performed. Figure 9, Figure 10, Figure 11, Figure 12 and Figure 13 shows 3D appearances in various types of MPS.

### 3.4. Salford Mucopolysaccharidosis Airway Score (SMAS)

The questionnaire looked at various aspects of the airway. Questions 1–10 in Table 2 reflect the upper airway, which is access. Questions 11–13 reflect mid airway, and questions 14 and 15 reflect the lower airways, which is the lung physiological functions.

#### Validation and Usefulness of SMAS

The scores were distributed to 25 senior anaesthetists in a tertiary hospital and were asked to use it in their daily practice. All the anaesthetists were requested to fill a questionnaire (Appendix A) that addressed content, criterion validity, and internal consistency. Following responses from the anaesthetic colleagues, we felt comfortable to use this in our study.

**Figure 14:**
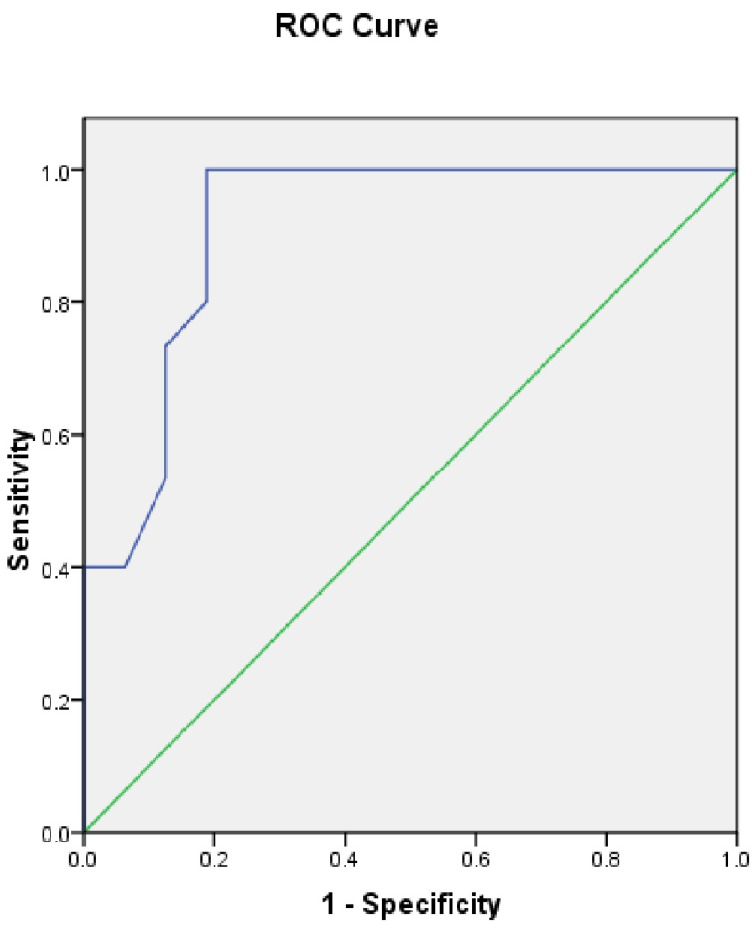
ROC curve to assess usefulness of SMAS as a predictor of complex airway.

All the 15 parameters were used in 21 patients. In four patients (MPS I = 1, MPS II = 1, MPS III = 2) pulmonary functions could not be done due to learning difficulties. In six patients (MPS I = 1, MPS II = 2, MPS IV = 2, MPS VII = 1), pulmonary functions were not considered as they were more than one year old. In the MPS III group (n = 2), cross-sectional imaging, 3D, and VE were not performed as they had no clinical symptoms of airway problems so they could only be assessed for 10 parameters. Table 5 summarises SMAS scores and percentages for all 31 adult MPS patients. It can be deduced that scores 0–15 (33%) correspond to mild airway abnormality, 15–30 (33–66.7%) to moderate abnormality, and 30–45 (66.8–100%) to severe abnormality. It was noted that patients in MPS II, MPS IV, and MPS VI groups have high scores. Patients who have scored more than 35 have had some form of airway intervention. Patient number 10, 14, and 30 needed tracheostomy to improve breathing. Patient number 11, 24, and 26 needed hospital admission for difficulty in breathing following a viral infection. All patients scoring more than 25 had obstructive sleep apnoea. Hypothetically, we can say that a score more than 25 should be considered a difficult airway and may have potential complications. To assess the sensitivity of this hypothesis, the receiver operating characteristic (ROC) was calculated. Patients who needed any airway intervention was the measurable outcome and the SMAS being the predictor. The sensitivity at score 25 is 1 and 0.9 at score 26. Figure 14 shows the ROC curve.

ROC—receiver operating characteristic, SMAS—Salford Mucopolysaccharidosis Airway Score.

It could be argued that a bulky airway may be secondary to body habitus. So Pearson’s correlation was used to assess the impact of height and body mass index on SMAS. It was noted that there was no statistically significant correlation noted. Table 6 summarises the correlation values.

## 4. Discussion

Holistic assessment of a difficult airway is key to successful management. It is important to assess and identify various abnormalities in a difficult airway; failure to identify these and failure to act on abnormal findings in the management of difficult airway can lead to poor outcomes [23]. The airway in MPS patients has a complex anatomy due to MPS deposits, skeletal, and soft tissue abnormalities. Adult MPS airway is complicated not just by age-related changes on the soft tissue and skeletal structures but also due to associated comorbidities. All the patients in our cohort had short stature, short necks, and restricted neck mobility, making access to the airway difficult. Large lower jaw in the MPS IV and MPS VI groups made access to the larynx difficult. The American Society of Anesthesiologists (ASA) [24] has considered the following outcomes as difficult airway—difficult facemask ventilation, difficult laryngoscopy, difficult tracheal intubation, failed intubation, and difficulty in placing and using supraglottic devices. In our experience, we have observed all of these in our adult MPS cohort in some form or other; all patients needed a smaller sized tube. We have found use of the microcuffed endotracheal tube produced by Avanos^®^ very useful in adult MPS airways. All of the difficult airway parameters pointed by ASA were noted in our MPS II group. MPS I tolerated supraglottic airway device, as the supraglottis was not bulky. In MPS IV and MPS VI groups, use of the supraglottic airway device was not considered due to large epiglottis and high anterior larynx. Roth et al. [25] reviewed 133 studies involving 844,206 participants to assess the diagnostic accuracy bedside tests for assessment of difficult airway. The common bedside tests to assess the difficult airway include Mallampati score [26], modified Mallampati score [11], Wilson score [27], thyromental distance, sternomental distance, mouth opening, and upper lip bite.

There is no one perfect tool for airway assessment; however, a combination of various tools is a better predictor of complex airway, and this is better than a single test used in isolation [28]. Cattano et al. [29] addressed the impact airway assessment by using 11 ASA’s airway risk factors by residents. The authors observed there was a better documentation of difficult airway but did not significantly impact the assessment of difficult airways. However, in the study group, there were only 17% of patients with difficult airways. It is not clear from the paper if the group had patients with a background of metabolic diseases, and the study used only bedside tests. By contrast, our group includes all patients with difficult airways with multiple comorbidities. In this new SMAS score developed by our team, not all parameters can be assessed in all patients, such as nasendoscopy and spirometry, which need the patient’s co-operation. This was the case in some MPS I and both MPS III patients with learning difficulties. In the same way, if the patient did not have any breathing or respiratory issues, such as MPS III, performing investigations will add undue discomfort to the patient. So, they should be only considered if the investigations change the way we manage our patients. Cross-sectional imaging using CT or MR scans are very useful. We have found assessment of the supraglottis is better with an MRI scan, as it shows the soft tissues better. The infraglottis, trachea, and lungs are better assessed by CT scans, as they show cartilaginous structures better and help in assessing the airway calibre, tortuosity, and malacia. Studies by Wittenborg et al. [30] in the 1960s suggested that a decrease in the diameter of 50% of the lumen of the trachea should be considered tracheomalacia. Tracheomalacia can be classified according to the reduction tracheal lumen into mild (50–75%), moderate (75–100%), and severe (100%), which is complete collapse [31].

Boiselle et al. [32] showed that tracheal collapse can be noted in expiration in healthy population; hence, tracheomalacia should be diagnosed in conjunction with clinical symptoms, signs, and lung functions. Although a CT scan may indicate changes suggestive of tracheomalacia, a dynamic expiratory CT scan demonstrates malacia better [33]. In our cohort of patients, tracheomalacia was also noted in CT scans taken in inspiration; hence, the actual problem of tracheomalacia in adult MPS may be underestimated. Symptomatic tracheomalacia can be conservatively managed by airway splinting using continuous positive air way pressure and surgically treated by stenting inside or outside the lumen of trachea, tracheostomy, and resection of the diseased segment of trachea [34] and tracheopexy [35]. Extrinsic compression of trachea by an anomalous innominate artery in a 16-year-old male with MPS IVa has been reported by Pizarro et al. [36]. The authors describe the surgical technique of tracheal and vascular reconstruction and have reported successful outcome without the need for tracheostomy. Any extensive thoracic surgery has to be carefully considered in adult MPS due to chest wall deformities, difficult airways, and associated comorbidities. Given the progressive nature of the disease, we managed our patients with a tracheostomy and positive pressure non-invasive ventilation rather than stenting and surgical splinting of the airway. We found the Bivona^®^ uncuffed adult tracheostomy tube from Smith medical^®^ very useful in our MPS II patient (patient number 14) with tracheomalacia. In one of our MPS VI patients (patient number 30), we managed tracheomalacia with tracheostomy and the paediatric Montgomery^®^ Safe-T-Tube™. The indication for tracheostomy in both these patients was worsening upper and lower airway obstruction, leading to obstructive sleep apnoea and dyspnoea at rest with resultant worsening quality of life. Tracheostomy was performed under a general anaesthetic following the securing of the upper airway with an endotracheal tube. Recovery following the tracheostomy in both these patients was protracted. Both were discharged following tracheostoma. There were no immediate complications, but stoma granulations were delayed complications needing topical corticosteroid ointments.

Respiratory function testing using spirometry is very useful in quantifying the lung physiology. However, obtaining a normalised value for the MPS patients will be difficult and will have to be interpreted carefully. Input from a respiratory physician with special interest in pulmonary physiology is important. It is important to consider that MPS are associated with both obstructive and restrictive airway disease. The use of spirometry is the most objective way of identifying these problems accepting practical limitations of use in certain situations. A six-minute walk test has been used as a surrogate of pulmonary function but is an overall functional assessment of combined cardiopulmonary status and therefore not specifically related to the airway’s disease. It is clear that in conditions associated with growth abnormalities that absolute values of FEV and FVC will be reduced. Therefore, the FEV and FVC percentages predicted are defined as the percentage predicted values based on age, sex, and height. The definition of obstruction is an FEV1 < 80% predicted and a FEV1/FVC ratio of <0.7. Conversely, a restrictive disease is reflected in a greater reduction in FVC compared with FEV1 resulting in a FEV1/FVC ration > 0.7. In the context of MPS, we have the obvious difficulty of the validity of height and age normalised curves in this group of patients. In the absence of alternatives, however, FEV1 and FVC will remain the mainstay of our monitoring of pulmonary functions. The value of spirometry is the ability to detect changes overtime, and it has been shown that there is a correlation with identification of sleep disordered breathing identified by the ODI 3% in MPS IVA [37]. A flow volume loop may help to assess intra- and extra thoracic obstruction, which will in turn aid in the management of the complex airway. However, in the absence of a normalised value, it may be difficult to interpret. We may also falsely interpret intra- and extra thoracic obstruction.

The SMAS was designed to quantify the degree of airway abnormality with an aim to assess all airway and breathing factors together. The aim was to use this score when adult MPS patients are planned for a general anaesthetic. Prominent teeth, mouth opening, neck movements, and modified Mallampati grade [26] are all important factors to access the airway. The thyromental angle and nasendoscopic findings help to decide if the larynx is high and/or anterior. Once the airway is accessed, the supraglottic, glottic, and subglottic bulkiness due to GAG deposits will dictate the size of the endotracheal tube. Once the airway is secured, the degree of tracheobronchomalacia and tracheal tortuosity will govern the oxygen delivery to lungs. We have scored tortuous trachea as 3, as this will have a big impact on securing the airway and ventilation. The pulmonary function tests will help us understand the physiological state of the lungs. In our experience, a score more than 25 indicates a complex airway. This information obtained during pre-operative assessment can be used to explain the patients, family members, and other health professionals to make a decision for general anaesthetic. We feel that a combination of parameters rather than a single parameter is useful in assessing the complex MPS airway. The limitations in our study are a small cohort of adult MPS patients; this is due to the rarity of the disease and reduced longevity. However, given the small cohort, extensive airway assessment has been performed. Secondly, SMAS cannot be scored for all parameters, especially in patients with learning difficulties; in this situation, percentage scores may be useful. Thirdly, the validation of the scores can be improved by performing on a larger cohort via a multicentre collaboration and perhaps assessing its usefulness in paediatric MPS. Haematopoietic stem cell transplantation (HSCT) [38] and enzyme replacement therapy (ERT) [39] have been shown to be effective therapeutic options for various types of MPS; they will reduce the deformities in adulthood, which in turn may lead to less complex airways. Future studies comparing patients who received therapy and those who did not will be helpful to understand the impact on skeletal and soft tissue abnormalities contributing to airway issues. Similarly, mutations of certain types may help us to prognosticate the severity of the airways. Unfortunately, both these could not be investigated in our study due to a small number of patients. A multicentre collaboration involving patients from various geographical regions may help in better understanding of the airway issues in this complex metabolic disease with multisystem involvement.

## 5. Conclusions

Adult MPS airway is challenging. With the advancements of treatment modalities, patients present with varying degrees of abnormalities in the airway. Given the rarity of the disease, mutations, and varying treatments in patients, it may be difficult to construe a specific airway pattern for each MPS. In our experience, we have noted that MPS I and III patients have milder airway abnormalities; MPS II patients have the most difficult airways; and MPS IV, VI, VII patients have both complex upper airway and tortuous trachea. Various factors have to be taken into consideration in assessing the complex airway with a multidisciplinary team. We have found use of the SMAS very helpful in assessing and quantifying the severity of airway problems. Adult MPS patients have complications associated with comorbidities, and communication issues due to vision and hearing. All these factors have to be carefully considered in assessing the complex airway. Nasendoscopy, cross-sectional imaging, 3D, VE, and respiratory functions are important tools apart from clinical examination in airway assessment. We recommend a joint ENT, anaesthetic, respiratory, and metabolic team with special interest in MPS for the assessment of adult MPS airway.

## Figures and Tables

**Table 1:** Various types of MPS; reproduced with permission from Braunlin et al. [5], who compiled data from Neufeld et al. [2] and Valayannopoulos et al. [6].

MPS Type (Eponym)	Incidence per 10^5^ Live Births; Inheritance Pattern	Typical Age at Diagnosis	Typical Life Expectancy If Untreated	Enzyme Deficiency	GAG
MPS I Hurler (H) MPS I Hurler–Scheie (H-S) MPS I Scheie (S)	0.11–1.67; AR	H: <1 year H-S: 3–8 years S: 10–20 years	H: death in childhood H-S: death in teens or early adulthood S: normal to slightly reduced lifespan	α-L-iduronidase	DS, HS
MPS II (Hunter)	0.1–1.07; XR	1–2 years when rapidly progressing	rapidly progressing: death < 15 years slowly progressing: death in adulthood	iduronate-2-sulfatase	DS, HS
MPS III (Sanfilippo) A-B-C-D	0.39–1.89; AR	4–6 years	death in puberty or early adulthood	heparan sulfamidase (A) N-acetyl-α-D-glucosaminidase (B) acetyl-CoA-α-glucosaminidase N-acetyltransferase (C) N-acetylglucosamine-6-sulfatase (D)	HS
MPS IV (Morquio) A-B	0.15–0.47; AR	1–3 years	death in childhood- middle age	N-acetylgalactosamine-6-sulfatase (A) β-galactosidase (B)	CS, KS (A) KS (B)
MPS VI (Maroteaux-Lamy)	0–0.38; AR	rapidly progressing: 1–9 years slowly progressing: >5 years	rapidly progressing: death in 2nd–3rd decade slowly progressing: death in 4–5th decade	N-acetylgalactosamine-4-sulfatase	DS
tblMPS VII (Sly)	0–0.29; AR	neonatal to adulthood	death in infancy- 4th decade **	β-D-glucuronidase	CS, DS, HS
MPS IX (Natowicz) *	unknown	adolescence	unknown	hyaluronidase	CS

AR—autosomal recessive, CS—chondroitin sulphate, DS—dermatan sulphate, GAG—glycosaminoglycan, H—Hurler syndrome, HS—heparan sulphate, H-S—Hurler–Scheie syndrome, KS—keratan sulphate, S—Scheie syndrome, XR—X-linked recessive. * Only 1 patient reported in literature (Natowicz et al. 1996); ** death can occur in utero with hydrops fetalis.

**Table 2:** Salford Mucopolysaccharidosis Airway Score (SMAS).

S. No.	Parameter	Measure	Score	Final Score
	MPS Type			
1	Mouth opening	>5 cm	0	
		4–5 cm	1	
		3–4 cm	2	
		<3 cm	3	
2	Teeth protrusion on clinical exam and scans	Non-protruding	0	
		Mild	1	
		Moderate	2	
		Severe	3	
3	Cervical spine mobility, stability	unrestricted	0	
		60–90 degrees flexion	1	
		30–60 degrees flexion	2	
		<30 degrees or unstable	3	
4	Tongue bulkiness on examination and Scan	Normal	0	
		Mild (filling less than 1/3 of floor mouth)	1	
		Moderate (filling 1/3 to 1/2 of oral cavity)	2	
		Severe (filling more than 1/2 of oral cavity)	3	
5	Modified Mallampati grade [11]	1	0	
		2	1	
		3	2	
		4	3	
6	Thyromental distance	>6 cm	0	
		5–6 cm	1	
		4–5 cm	2	
		<4 cm	3	
7	Larynx height epiglottis to soft palate	>4 cm	0	
		3–4 cm	1	
		2–3 cm	2	
		<2 cm	3	
8	Epiglottis bulkiness	Normal (filling less than 1/3 of oropharynx)	0	
		Mild (filling 1/3 to 1/2 of oropharynx)	1	
		Moderate (filling 1/2 to complete oropharynx)	2	
		Severe (Filling entire oropharynx)	3	
9	Supraglottis bulkiness	Normal (filling less than 1/3 of laryngopharynx)	0	
		Mild (filling 1/3 to 1/2 of laryngopharynx)	1	
		Moderate (filling ½ to complete laryngopharynx)	2	
		Severe (filling entire oropharynx)	3	
10	Glottis bulkiness	Normal (filling less than 1/3 of glottis)	0	
		Mild (filling 1/3 to 1/2 of glottis)	1	
		Moderate (filling 1/2 to complete glottis)	2	
		Severe (filling entire glottis)	3	
11	Subglottis diameter at cricoid level	>7 mm	0	
		6–7 mm	1	
		5–6 mm	2	
		<5 mm	3	
12	Tracheomalacia or tracheal stenosis (degree of narrowing)	No narrowing	0	
		50–75% lumen narrowing	1	
		75–99% lumen narrowing	2	
		100% lumen narrowing	3	
13	Tracheal tortuosity	None	0	
				
				
		present	3	
14	FEV1%	>80%	0	
		60–79%	1	
		40–59%	2	
		<40%	3	
15	FVC%	>80%	0	
		60–79%	1	
		40–59%	2	
		<40%	3	

**Table 3:** Demographics of all adult MPS patients.

MPS Type	Number	Age in Years and Number of Patients	Sex
1	9	23–27 = 5 31–32 = 2 39 = 1 43 = 1	Females = 3 Males = 6
2	12	20–27 = 7 29–33 = 5	All males
3	2	19–21 = 2	Both females
4	5	19, 21, 23, 33, 41	Females = 4 Male = 1
6	2	27, 38	Female = 1, Male = 1
7	1	31	Male

**Table 4:** Common airway abnormalities and relevant airway challenges in each type of adult MPS.

MPS Type	Airway Findings	Relevant Airway Challenges
1	Protruding teeth, large tongue, high larynx, moderately bulky supraglottis, narrow subglottis	Learning difficulties, poor vision
2	Protruding teeth, large tongue, high larynx, very bulky supraglottis, narrow subglottis, tracheobronchomalacia	Cervical spine instability, short neck
3	Large tongue, malacia of supraglottis	Learning difficulties
4	Large tongue, protruding teeth, large jaw, moderately bulky supraglottis, large epiglottis, tortuous trachea with tracheomalacia	Short neck, hypermobility
6	Large tongue, protruding teeth, large jaw, large epiglottis, tortuous trachea with tracheomalacia	Short neck, c-spine problems, kyphoscoliosis
7	Slightly protruding teeth, high larynx, narrow subglottis, tortuous trachea	Kyphoscoliosis

MPS—mucopolysacharridosis.

**Table 5:** Summary of the SMAS scores for all patients, mutation type, and therapy.

Patient Number	MPS Type	Sex	Age in Years	SMAS	Score Percentage	Associated Abnormality	Therapy	Mutation
1	I	M	39.1	20	51.3		HSCT	p.(Trp402Ter), p.(Leu218Pro)
2	I	M	31.3	15	33.3		HSCT	p.(Leu490Pro), p.(Leu490Pro)
3	I	M	25.9	23	51.1		HSCT	p.(Trp402Ter), p.(Trp402Ter)
4	I	M	25.7	10	22.2		HSCT	p.(Gln70Ter), p.(Gln70Ter)
5	I	M	32.2	24	53.3		HSCT	p.(Trp402Ter), p.(Ser633Leu)
6	I	M	26.7	31	68.9	OSA	ERT	hom L490P, exon 10 IDUA gene
7	I	F	23.0	10	22.2		ERT	N/A
8	I	F	43.0	29	64.4	OSA	ERT	N/A
9	I	F	24.0	20	48.7		ERT	N/A
10	II	M	30.1	40	88.9	TRACH	ERT	c.1152delT, exon 8 IDS gene
11	II	M	31.7	36	80	OSA	ERT	N/A
12	II	M	29.1	35	77.8	OSA	ERT	N/A
13	II	M	20.7	32	82.1	OSA	ERT	c.1528insT
14	II	M	33.0	40	88.9	TRACH	ERT STOPPED	N/A
15	II	M	32.9	34	75.6	OSA	ERT	N/A
16	II	M	25.2	32	82.1	OSA	ERT	N/A
17	II	M	26.3	11	24.4		ERT	T14gT, exon4 IDS gene
18	II	M	23.7	21	46.7		ERT	N/A
19	II	M	20.9	31	79.5	OSA	ERT	N/A
20	II	M	26.9	29	64.4	OSA	ERT	missense mutation in N63D, exon 2, IDS gene
21	II	M	23.3	14	31.1		ERT	N/A
22	III	F	19.6	9	30		none	N/A
23	III	F	20.3	2	6.7		none	hom c.234+1G>T, HGSNAT gene
24	IV	F	23.0	32	82.1	OSA	none	N/A
25	IV	F	21.1	28	71.7	OSA	ERT?	N/A
26	IV	F	41.1	38	84.4	OSA	ERT STOPPED	hom c.871G>A p.(Ala291Thr) GALNS gene
27	IV	M	33.5	27	60	OSA	none	N/A
28	IV	M	19.3	26	57.8	OSA	ERT	N/A
29	VI	F	27.3	30	66.7	OSA	ERT	T442R/245delT
30	VI	M	38.1	41	91.1	TRACH	ERT paused	N/A
31	VII	M	31.0	16	41.1		none	c.526C>T p.(Leu176Phe), c.1820G>C p.(Gly607Ala)

MPS—mucopolysacharridosis, M—male, F—female, HSCT—haematopoetic stem cell transplantation, ERT—enzyme replacement therapy, SMAS—Salford Metabolic Airway Score, TRACH—tracheostomy, OSA—obstructive sleep apnoea.

**Table 6:** Pearson’s correlation between Salford Metabolic Airway Score and height, weight, and body mass index.

Subtitle	Subtitle	Total Score
Height	Correlation coefficient	−0.438
	*p*-value	0.014
Weight	Correlation coefficient	−0.168
	*p*-value	0.367
Body Mass Index	Correlation coefficient	0.340
	*p*-value	0.061

## Data Availability

All the necessary medical information has been provided in the study. Any further data presented in this study are available on request from the corresponding author. The data are not publicly available due to privacy.

## Appendix A. Difficult Airway Assessment Questionnaire

Dear Colleague,

Your feedback is very important to us.

Can you please give your opinion (agree or disagree) on 15 parameters which can be used to assess a difficult airway, breathing, respiration assuming you have all investigations such as clinical examination, nasendoscopy, and CT scans available.

There are only 16 questions, which will take less than 5 min to answer.

Many thanks for your opinion.

Chai Gadepalli

1. Mouth opening.

More than 5 cm = 0 score
4–5 cm = 1
3–4 cm = 2
Less than 3 cm = 3

Strongly agree
Agree
Neutral
Disagree
Strongly disagree

---------------------------------

2. Teeth protrusion.

Non-protruding = 0 score
Mild protrusion = 1
Moderate protrusion = 2
Severe protrusion = 3 *

Strongly agree
Agree
Neutral
Disagree
Strongly disagree

---------------------------------

3. Cervical spine mobility, stability on clinical examination.

Unrestricted = 0 score
60–90 degrees flexion = 1
30–60 degrees flexion = 2
Less than 30 degrees flexion or unstable or fixed = 3 *

Strongly agree
Agree
Neutral
Disagree
Strongly disagree

---------------------------------

4. Tongue bulkiness on examination and scan.

Normal = 0 score
Mild = 1 (tongue fills floor of the mouth)
Moderate = 2 (tongue fills between 1/3 to 1/2 of oral cavity)
Severe = 3 (tongue fills more than 1/2 of oral cavity) *

Strongly agree
Agree
Neutral
Disagree
Strongly disagree

---------------------------------

5. Modified Mallampati grade.

Grade 1 = 0 score
Grade 2 = 1
Grade 3 = 2
Grade 4 = 3 *

Strongly agree
Agree
Neutral
Disagree
Strongly disagree

---------------------------------

6. Thyromental distance on clinical examination.

More than 6 cm = 0 score
5–6 cm = 1
4-5 cm = 2
Less than 4 cm = 3 *

Strongly agree
Agree
Neutral
Disagree
Strongly disagree

---------------------------------

7. Larynx height-distance between epiglottis and soft palate on nasendoscopy/scan.

More than 4 cm = 0 score
3–4 cm = 1
2–3 cm = 2
Less than 2 cm = 3

Strongly agree
Agree
Neutral
Disagree
Strongly disagree

---------------------------------

8. Epiglottis bulkiness on nasendoscopy / scan.

Normal= 0 score (filling less than less than 1/3 of oropharynx)
Mild bulkiness = 1 (filling 1/3–1/2 of oropharynx)
Moderate bulkiness = 2 (filling 1/2 to complete oropharynx)
Severe bulkiness = 3 (filling the entire oropharynx) *

Strongly agree
Agree
Neutral
Disagree
Strongly disagree

---------------------------------

9. Supraglottis bulkiness on nasendoscopy/scan.

Normal= 0 score (filling less than less than 1/3 of supraglottis)
Mild bulkiness = 1 (filling 1/3–1/2 of supraglottis)
Moderate bulkiness = 2 (filling 1/2 to complete supraglottis)
Severe bulkiness = 3 (filling the entire supraglottis) *

Strongly agree
Agree
Neutral
Disagree
Strongly disagree

---------------------------------

10. Glottis bulkiness on nasendoscopy.

Normal = 0 score (filling less than less than 1/3 of glottis)
Mild bulkiness = 1 (filling 1/3–1/2 of glottis)
Moderate bulkiness = 2 (filling 1/2 to complete glottis)
Severe bulkiness = 3 (filling the entire glottis) *

Strongly agree
Agree
Neutral
Disagree
Strongly disagree

---------------------------------

11. Subglottis diameter at cricoid level on CT scan.

More than 7 mm = 0 score
6–7 mm = 1
5–6 mm = 2
Less than 5 mm = 3 *

Strongly agree
Agree
Neutral
Disagree
Strongly disagree

---------------------------------

12. Tracheomalacia or stenosis (degree of lumen collapse) on CT scan.

No malacia = 0 score
50–75% lumen collapse = 1
75–99% lumen collapse = 2
100% lumen collapse = 3 *

Strongly agree
Agree
Neutral
Disagree
Strongly disagree

---------------------------------

13. Tracheal tortuosity on CT scan.

None = 0 score
Tortuosity present = 3

Strongly agree
Agree
Neutral
Disagree
Strongly disagree

---------------------------------

14. FEV1% in last one year.

More than > 80% = 0 score
60–79% = 1
40–59% = 2
Less than 40% = 3

Strongly agree
Agree
Neutral
Disagree
Strongly disagree

---------------------------------

15. FVC% in last one year.

More than 80% = 0 score
60–79% = 1
40–59% = 2
Less than 40% = 3 *

Strongly agree
Agree
Neutral
Disagree
Strongly disagree

---------------------------------

16. The above questions helps me in holistic airway assessment (contains all criteria to assess a difficult airway) *

Strongly agree
Agree
Neutral
Disagree
Strongly disagree

---------------------------------

17. Please use the space below to make any comments

---------------------------------End of questionnaire---------------------------------

## References

[1] Mehta A.B., Winchester B. (2012). Lysosomal Storage Disorders: A Practical Guide.

[2] Neufeld E., Muenzer J., Scriver C.R., Beaudet A.L., Sly W.S., Valle D., Childs R., Kinzler K.W. (2001). The Mucopolysaccharidoses. The Metabolic and Molecular Bases of Inherited Diseases.

[3] Muenzer J. (2011). Overview of the mucopolysaccharidoses. Rheumatology.

[4] Clark B.M., Sprung J., Weingarten T.N., Warner M.E. (2018). Anesthesia for patients with mucopolysaccharidoses: Comprehensive review of the literature with emphasis on airway management. Bosn. J. Basic Med. Sci..

[5] Braunlin E.A., Harmatz P.R., Scarpa M., Furlanetto B., Kampmann C., Loehr J.P., Ponder K.P., Roberts W.C., Rosenfeld H.M., Giugliani R. (2011). Cardiac disease in patients with mucopolysaccharidosis: Presentation, diagnosis and management. J. Inherit. Metab. Dis..

[6] Valayannopoulos V., Nicely H., Harmatz P., Turbeville S. (2010). Mucopolysaccharidosis vi. Orphanet J. Rare Dis..

[7] Berger K.I., Fagondes S.C., Giugliani R., Hardy K.A., Lee K.S., McArdle C., Scarpa M., Tobin M.J., Ward S.A., Rapoport D.M. (2013). Respiratory and sleep disorders in mucopolysaccharidosis. J. Inherit. Metab. Dis..

[8] Muhlebach M.S., Wooten W., Muenzer J. (2011). Respiratory manifestations in mucopolysaccharidoses. Paediatr. Respir. Rev..

[9] Tsara V., Amfilochiou A., Papagrigorakis J., Georgopoulos D., Liolios E., Kadiths A., Koudoumnakis E., Aulonitou E., Emporiadou M., Tsakanikos M. (2010). Guidelines for diagnosing and treating sleep related breathing disorders in adults and children (Part 3: Obstructive sleep apnea in children, diagnosis and treatment). Hippokratia.

[10] Dalewski B., Kamińska A., Syrico A., Kałdunska A., Pałka Ł., Sobolewska E. (2021). The Usefulness of Modified Mallampati Score and CT Upper Airway Volume Measurements in Diagnosing OSA among Patients with Breathing-Related Sleep Disorders. Appl. Sci..

[11] Huang H.-H., Lee M.-S., Shih Y.-L., Chu H.-C., Huang T.-Y., Hsieh T.-Y. (2011). Modified Mallampati classification as a clinical predictor of peroral esophagogastroduodenoscopy tolerance. BMC Gastroenterol..

[12] Thurtell M.J., Bruce B.B., Rye D.B., Newman N.J., Biousse V. (2011). The Berlin questionnaire screens for obstructive sleep apnea in idiopathic intracranial hypertension. J. Neuro-Ophthalmol..

[13] Wraith J.E. (2005). The first 5years of clinical experience with laronidase enzyme replacement therapy for mucopolysaccharidosis I. Expert Opin. Pharmacother..

[14] Wraith J.E., Beck M., Lane R., Van Der Ploeg A., Shapiro E., Xue Y., Kakkis E.D., Guffon N. (2007). Enzyme replacement therapy in patients who have mucopolysaccharidosis I and are younger than 5 years: Results of a multinational study of recombinant human α-L-iduronidase (laronidase). Pediatrics.

[15] Hendriksz C.J., Giugliani R., Harmatz P., Mengel E., Guffon N., Valayannopoulos V., Parini R., Hughes D., Pastores G.M., Lau H.A. (2015). Multi-domain impact of elosulfase alfa in Morquio A syndrome in the pivotal phase III trial. Mol. Genet. Metab..

[16] Hendriksz C.J., Burton B., Fleming T.R., Harmatz P., Hughes D., Jones S.A., Lin S.-P., Mengel E., Scarpa M., Valayannopoulos V. (2014). Efficacy and safety of enzyme replacement therapy with BMN 110 (elosulfase alfa) for Morquio A syndrome (mucopolysaccharidosis IVA): A phase 3 randomised placebo-controlled study. J. Inherit. Metab. Dis..

[17] Clarke L.A., Wraith J.E., Beck M., Kolodny E.H., Pastores G.M., Muenzer J., Rapoport D.M., Berger K.I., Sidman M., Kakkis E.D. (2009). Long-term efficacy and safety of laronidase in the treatment of mucopolysaccharidosis I. Pediatrics.

[18] Harmatz P., Giugliani R., Schwartz I.V.D., Guffon N., Teles E.L., Miranda M.C.S., Wraith J.E., Beck M., Arash L., Scarpa M. (2008). Long-term follow-up of endurance and safety outcomes during enzyme replacement therapy for mucopolysaccharidosis VI: Final results of three clinical studies of recombinant human *N*-acetylgalactosamine 4-sulfatase. Mol. Genet. Metab..

[19] Aldenhoven M., Wynn R.F., Orchard P.J., O’Meara A., Veys P., Fischer A., Valayannopoulos V., Neven B., Rovelli A., Prasad V.K. (2015). Long-term outcome of Hurler syndrome patients after hematopoietic cell transplantation: An international multicenter study. Blood J. Am. Soc. Hematol..

[20] Arn P., Bruce I.A., Wraith J.E., Travers H., Fallet S. (2015). Airway-related symptoms and surgeries in patients with mucopolysaccharidosis I. Ann. Otol. Rhinol. Laryngol..

[21] Pal A.R., Mercer J., Jones S.A., Bruce I.A., Bigger B.W. (2018). Substrate accumulation and extracellular matrix remodelling promote persistent upper airway disease in mucopolysaccharidosis patients on enzyme replacement therapy. PLoS ONE.

[22] Kirkpatrick K., Ellwood J., Walker R.W. (2012). Mucopolysaccharidosis type I (Hurler syndrome) and anesthesia: The impact of bone marrow transplantation, enzyme replacement therapy, and fiberoptic intubation on airway management. Pediatric Anesth..

[23] Katz J.A., Avram M.J. (2012). 4th National Audit Project of the Royal College of Anaesthetists and The Difficult Airway Society: Major Complications of Airway Management in the United Kingdom: Report and Findings. J. Am. Soc. Anesthesiol..

[24] Apfelbaum J., Hagberg C., Caplan R., Blitt C., Connis R., Nickinovich D., Benumof J., Berry F. (2013). American Society of Anesthesiologists Task Force on Management of the Difficult Airway Practice guidelines for management of the difficult airway: An updated report by the American Society of Anesthesiologists Task Force on Management of the Difficult Airway. Anesthesiology.

[25] Roth D., Pace N., Lee A., Hovhannisyan K., Warenits A., Arrich J., Herkner H. (2019). Bedside tests for predicting difficult airways: An abridged Cochrane diagnostic test accuracy systematic review. Anaesthesia.

[26] Mallampati S.R., Gatt S.P., Gugino L.D., Desai S.P., Waraksa B., Freiberger D., Liu P.L. (1985). A clinical sign to predict difficult tracheal intubation; a prospective study. Can. Anaesth. Soc. J..

[27] Wilson M., Spiegelhalter D., Robertson J., Lesser P. (1988). Predicting difficult intubation. BJA Br. J. Anaesth..

[28] Crawley S., Dalton A. (2015). Predicting the difficult airway. BJA Educ..

[29] Cattano D., Killoran P., Iannucci D., Maddukuri V., Altamirano A., Sridhar S., Seitan C., Chen Z., Hagberg C. (2013). Anticipation of the difficult airway: Preoperative airway assessment, an educational and quality improvement tool. Br. J. Anaesth..

[30] Wittenborg M., Gyepes M., Crocker D. (1967). Tracheal dynamics in infants with respiratory distress, stridor, and collapsing trachea. Radiology.

[31] Murgu S.D., Colt H.G. (2007). Description of a multidimensional classification system for patients with expiratory central airway collapse. Respirology.

[32] Boiselle P.M., O’Donnell C.R., Bankier A.A., Ernst A., Millet M.E., Potemkin A., Loring S.H. (2009). Tracheal collapsibility in healthy volunteers during forced expiration: Assessment with multidetector CT. Radiology.

[33] Baroni R.H., Feller-Kopman D., Nishino M., Hatabu H., Loring S.H., Ernst A., Boiselle P.M. (2005). Tracheobronchomalacia: Comparison between end-expiratory and dynamic expiratory CT for evaluation of central airway collapse. Radiology.

[34] Murgu S.D., Colt H.G. (2006). Treatment of adult tracheobronchomalacia and excessive dynamic airway collapse. Treat. Respir. Med..

[35] Svetanoff W.J., Jennings R.W. (2018). Updates on surgical repair of tracheobronchomalacia. J. Lung Health Dis..

[36] Pizarro C., Davies R.R., Theroux M., Spurrier E.A., Averill L.W., Tomatsu S. (2016). Surgical reconstruction for severe tracheal obstruction in Morquio A syndrome. Ann. Thorac. Surg..

[37] Kenth J.J., Thompson G., Fullwood C., Wilkinson S., Jones S., Bruce I. (2019). The characterisation of pulmonary function in patients with mucopolysaccharidoses IVA: A longitudinal analysis. Mol. Genet. Metab. Rep..

[38] Taylor M., Khan S., Stapleton M., Wang J., Chen J., Wynn R., Yabe H., Chinen Y., Boelens J.J., Mason R.W. (2019). Hematopoietic stem cell transplantation for mucopolysaccharidoses: Past, present, and future. Biol. Blood Marrow Transplant..

[39] Concolino D., Deodato F., Parini R. (2018). Enzyme replacement therapy: Efficacy and limitations. Ital. J. Pediatrics.

